# A rare case of simultaneous spontaneous coronary artery dissections in the circumflex and the right coronary arteries presenting as acute myocardial infarction: a case report

**DOI:** 10.11604/pamj.2024.49.109.42191

**Published:** 2024-12-04

**Authors:** Fekher Jaoued, Zied Ben Ameur, Wassim Saoudi, Hedi Frigui, Raouf Kchaou, Wissal Rouatbi, Mohamed Aymen Ben Abdesslam, Samia Ernez Hajri

**Affiliations:** 1 Department of Cardiology, Farhat Hached Hospital, Sousse, Tunisia

**Keywords:** Coronary artery dissection, myocardial infarction, coronary angiography, case report

## Abstract

Simultaneous Spontaneous Coronary Artery Dissection (SCAD) involving multiple coronary arteries is an exceptionally rare occurrence. We present a unique case of a 55-year-old woman with no known cardiovascular risk factors who presented with severe chest pain and ST-segment elevation myocardial infarction (STEMI). Coronary angiography revealed simultaneous SCAD in both the Right Coronary Artery (RCA) and the Circumflex (Cx) artery, an unprecedented finding. The patient underwent percutaneous coronary intervention (PCI) for the RCA and received medical therapy for the Cx artery. The three-month follow-up demonstrated the disappearance of SCAD in the Cx artery and patent stents in the RCA, indicating positive patient outcomes. This case highlights the complexity of SCAD and the importance of individualized treatment strategies.

## Introduction

Spontaneous Coronary Artery Dissection (SCAD) is an uncommon yet increasingly recognized cause of acute coronary syndromes (ACS), which particularly affects women with few or no traditional cardiovascular risk factors [1]. It is characterized by the intimal tear of a coronary artery, leading to the formation of an intramural hematoma that can compromise blood flow [1]. Although SCAD is relatively rare, the simultaneous occurrence of SCAD in multiple coronary arteries within the same patient is an exceedingly unusual phenomenon.

We report a rare case of simultaneous SCAD in both the left Circumflex (Cx) and right coronary arteries (RCA) of a middle-aged woman presented with an acute myocardial infraction (AMI).

## Patient and observation

**Patient information:** a 55-year-old woman, with no previous medical history or known cardiovascular risk factors, presented with severe chest pain (visual analog scale score of 9/10) at rest persisting for 1 hour. Her symptoms were accompanied by nausea and vomiting. The patient had no history of cardiovascular risk factors, such as smoking or drug consumption, and no family history of sudden death or coronary artery disease. She was menopausal and didn´t take any hormonal therapies.

**Clinical findings:** upon admission, the patient was conscious, with a heart rate of 70 beats per minute. She experienced a hypertensive peak with blood pressure reaching 190/80 mmHg. There were no clinical signs of heart failure or peripheral signs of shock.

**Figure 1:**
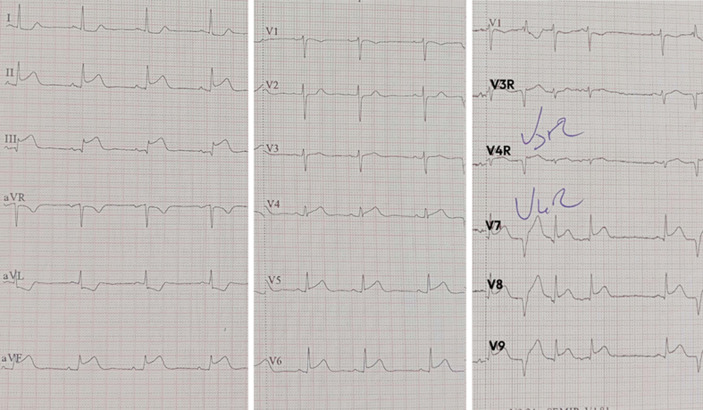
initial electrocardiogram showing a sinus rhythm with a normal axis, narrow QRS, and 0.3 mV ST-segment elevation in the inferior and posterior leads (DII, DIII, AvF, V7, V8, and V9)

**Diagnostic assessment:** her initial electrocardiogram (ECG) revealed a sinus rhythm with a normal axis, a narrow QRS complex, and 0.3 mV ST-segment elevation in the inferior and posterior leads (DII, DIII, AvF, V7, V8, and V9) (Figure 1). Given her symptoms and ECG findings, an acute inferior ST-segment elevation myocardial infarction (STEMI) was suspected, and the patient was promptly transferred to the cardiology department for primary angioplasty within 1 hour and a half of symptom onset.

**Figure 2:**
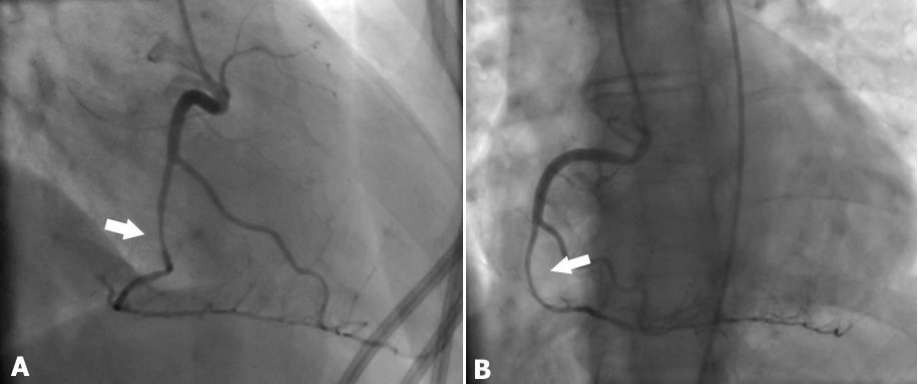
coronary angiography showing a severe reduction in the lumen of the right coronary artery (RCA) (arrow) from its second segment to the distal RCA due to spontaneous coronary artery dissection (arrow): (A) right anterior oblique projection; (B) left anterior oblique projection

**Figure 3:**
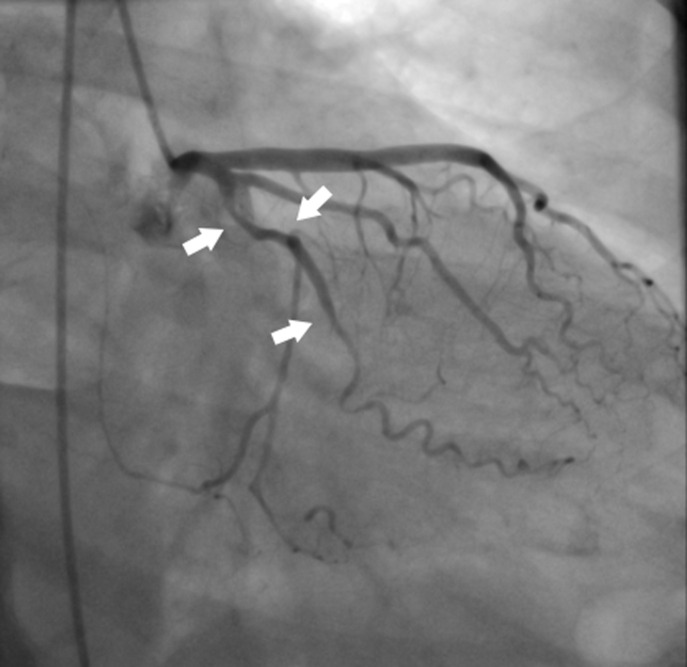
right oblique anterior projection showing spontaneous coronary dissection (arrows) of the circumflex artery extending to the marginal artery

A coronary angiography was performed. Firstly, it revealed a severe significant reduction in the lumen of the RCA, extending from its 2nd segment to its distal portion (Figure 2). This reduction was attributed to a coronary dissection that resulted in delayed coronary antegrade flow, suggesting that the RCA was the infract-related coronary artery. Additionally, her Left Main Coronary Artery (LMCA) and Left Anterior Descending Artery (LAD) exhibited a smooth-walled artery with no evidence of atherosclerosis; however, the Cx showed a coronary dissection extending up to the marginal artery significantly reducing the artery´s lumen (Figure 3).

**Figure 4:**
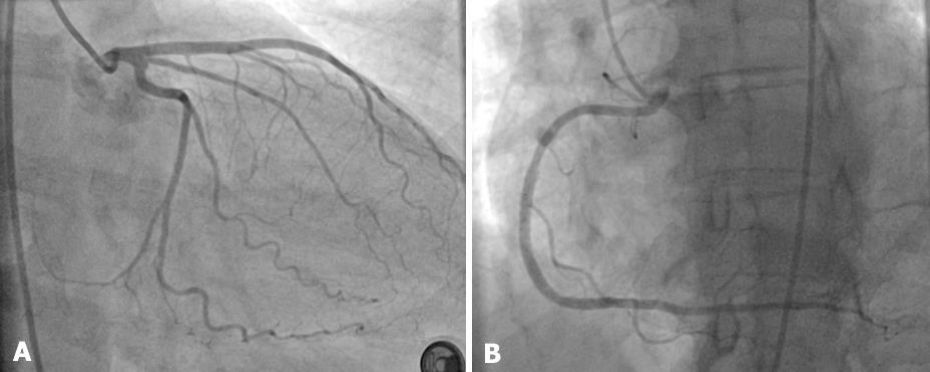
control coronary angiogram showing the disappearance of the circumflex dissection with smooth coronary arteries (A) and the patency of the right coronary artery stents (B)

**Diagnosis:** simultaneous SCAD of both RCA and Cx arteries was confirmed.

**Therapeutic interventions:** due to persistent chest pain and the persistence of ST-segment elevation in the inferior and posterior leads, the patient underwent angioplasty of the distal portion of the RCA using a drug-eluting stent of 2.75 x 40 mm to cover the entire SCAD. However, this procedure was complicated by the extension of SCAD into the second segment of the RCA, requiring the deployment of another drug-eluting stent of 2.5 x 23 mm. Subsequent angiographic control demonstrated the restoration of normal antegrade coronary flow and complete sealing of the SCAD. Following the intervention, the patient's chest pain symptoms resolved completely, and the ST-segment elevation on the ECG normalized. The decision was made to schedule the patient for a repeat coronary angiogram and she was discharged on dual antiplatelet therapy, including clopidogrel and aspirin.

**Follow-up and outcome of interventions:** after a three-month follow-up, the patient remained free of symptoms, and a control coronary angiogram showed the disappearance of the SCAD in the Cx artery and patent stents in the RCA artery (Figure 4).

**Patient's perspective:** the patient sought urgent care due to severe chest pain, nausea, and vomiting. Her experience likely involved anxiety and a desire for a rapid resolution to her distressing symptoms.

**Informed consent:** it was obtained from the patient before any procedures or interventions. The purpose, risks, and potential benefits were explained, and the patient consented to the recommended treatments.

## Discussion

This case report highlights a unique and challenging presentation of simultaneous SCAD in both the RCA and the Cx in a patient with no known cardiovascular risk factors. SCAD is an uncommon yet increasingly recognized cause of ACS, particularly in young and middle-aged women. While SCAD is relatively rare, its prevalence has been steadily increasing, prompting heightened awareness and prompt diagnosis among healthcare providers [1,2]. It is characterized by an intimal tear that allows blood to dissect between the intima and media or between the media and adventitia, leading to the formation of an intramural hematoma. Unlike traditional atherosclerotic coronary artery disease, SCAD typically affects the media or adventitia layers of the coronary artery rather than the intima [2].

The presentation of SCAD can vary widely, ranging from asymptomatic to severe chest pain, shortness of breath, and even sudden cardiac death [3]. A study by Johnson *et al*. (2021) found that the most common presenting symptoms of SCAD were chest pain (88%), shortness of breath (38%), and nausea/vomiting (29%) [3]. Furthermore, about 39% of SCAD patients presenting with ST-segment elevation on the ECG, a crucial finding that suggests myocardial infarction [3]. This is in line with the case discussed in our report, where ST-segment elevation on the ECG was a pivotal factor in diagnosing the AMI associated with simultaneous SCAD.

The diagnosis of SCAD is typically made based on coronary angiography, which reveals the characteristic intimal tear and intramural hematoma [2]. While the presence of an intimal tear and an intramural hematoma on coronary angiography is highly indicative of SCAD, the angiographic classification of SCAD further guides treatment decisions. The Yip-Saw angiographic classification system, proposed by Yip *et al*. [4], divides SCAD into three types based on the angiographic appearance of the dissection: type 1 SCAD characterized by multiple lumens and extra-luminal contrast staining, type 2 SCAD which appears as long, smooth stenosis, as in our case, and type 3 SCAD which mimics atherosclerosis and requires intracoronary imaging for definitive diagnosis [4].

While coronary angiography remains the primary diagnostic tool for SCAD, it can sometimes fail to detect subtle dissection features, leading to underdiagnosis. In fact, recent advancements in intravascular ultrasound (IVUS) and optical coherence tomography (OCT) imaging have significantly enhanced the accuracy of the diagnosis, particularly in cases where angiographic findings are inconclusive [5]. It´s worth noting that in patients with a certain diagnosis of SCAD based on angiography and a planned medical therapy, the use of intravascular imaging is generally not recommended due to safety considerations [5].

Spontaneous Coronary Artery Dissection (SCAD) typically manifests as a single-vessel disease with a predilection for the LAD artery, followed by the RCA, the LMCA and the Cx artery. However, the simultaneous occurrence of SCAD in both the RCA and Cx, as observed in this case, is exceptionally rare [6]. Few cases have previously reported the simultaneous occurrence of SCAD involving the left coronary artery extending to the LAD and Cx arteries. To the best of our knowledge, our case represents not only the second instance of SCAD involving the RCA and the left coronary artery [7] but also the first reported case of SCAD specifically affecting the Cx and RCA arteries.

Treatment for SCAD is typically conservative, with antiplatelet therapy and beta-blockers [2]. However, percutaneous coronary intervention (PCI) or coronary artery bypass grafting (CABG) may be necessary in some cases, particularly for patients with ongoing myocardial ischemia and reduced antegrade flow. According to the European Society of Cardiology (ESC), PCI is recommended only for patients with SCAD who have symptoms, signs of ongoing myocardial ischemia, or reduced antegrade flow [8], such as our patient who presented with chest pain and delayed antegrade flow in the RCA.

However, PCI in SCAD patients carries a higher risk of complications compared to PCI in patients with atherosclerosis-related coronary artery disease. The stent deployment in the RCA's SCAD was complicated by the extension of the dissection to its second segment. An international case series found that coronary complications following PCI occurred in over 30% of SCAD patients [9]. This increased risk is attributed to the unique pathophysiology of SCAD, in which the hematoma can extend and lead to further dissection when a stent is deployed.

A number of potential triggers have been identified in up to two-thirds of SCAD patients, with extreme physical or emotional stress being the most common [2]. Among SCAD patients who report precipitating factors, emotional stressors appear to be more common in women, while physical stressors have been reported more often in men [2]. Interestingly, our patient presented with a hypertensive peak upon admission, which could potentially be correlated with the occurrence of SCAD. However, the exact mechanisms underlying SCAD remain poorly understood, making it difficult to predict its occurrence and guide treatment decisions. The prognosis for SCAD is generally good and medical therapy can be safely applied to the majority of patients [9]. However, SCAD can recur in up to 20% of patients [10].

This case is interesting and unique in many ways. First, the simultaneous occurrence of SCAD in both RCA and Cx arteries is extremely rare. This occurrence is exceptionally rare, and to the best of our knowledge, no prior case has been reported showing such a simultaneous event. Second, the presentation of this case as a STEMI in a patient without traditional risk factors is of particular interest. Third, the diverse treatment strategies chosen for this case are another aspect that sets it apart. The decision, guided by clinical and angiographic findings, to employ medical treatment for one coronary artery and PCI for the other highlights this difference. Finally, the case report provides an opportunity to assess the outcomes of the two different treatment approaches employed. However, this is a single-case report, making findings non-generalizable. Additionally, while the diagnostic and treatment details are well-documented, the limited three-month follow-up underscores the importance of longer-term assessments for a comprehensive understanding of intervention durability and patient prognosis.

## Conclusion

This unique case of SCAD affecting both the RCA and the Cx arteries shows the remarkable complexity of this condition. In women presenting with STEMI without cardiovascular risk factors, SCAD should be considered. Successful management through PCI and medical therapy, along with positive follow-up results, suggests the potential for favorable outcomes when tailored approaches are employed.
